# Comparative efficacy of different durations and dosages of vonoprazan and amoxicillin dual therapy: a network meta-analysis of randomized controlled trials

**DOI:** 10.3389/fmed.2026.1853849

**Published:** 2026-08-28

**Authors:** Feng-yi Wei, Zhen-peng Huang, Sha Nie, Ru-jie Gao

**Affiliations:** Faculty of Nursing, Guangxi University of Chinese Medicine, Nanning, China

**Keywords:** amoxicillin, dosing regimen, eradication rate, *Helicobacter pylori*, treatment optimization, vonoprazan

## Abstract

**Background:**

The combination of vonoprazan and amoxicillin (VA) has been shown to be effective in eradicating *Helicobacter pylori* infection; however, the optimal treatment duration and dosage are yet to be determined.

**Methods:**

A systematic literature search of PubMed, Embase, and China National Knowledge Infrastructure was performed to identify randomized controlled trials (RCTs) comparing different durations of VA dual therapy or comparing this dual therapy with bismuth-containing quadruple therapy (BQT). All trials were conducted in China. A Bayesian network meta-analysis was used to evaluate the eradication rates and incidence of adverse events across different treatment regimens.

**Results:**

Thirty-eight randomized controlled trials were ultimately included in the analysis. Network meta-analysis revealed that both the 14- and 10-day VA regimens achieved higher eradication rates compared with BQT, with odds ratios (OR) of 1.7 [*95% credible interval* (*CrI*) 1.3–2.1] and 1.4 (*95% CrI* 1.1–2.0), respectively. The 7-day regimen did not yield a difference in eradication rate compared with BQT (*P* > 0.05). All VA dual therapy regimens were associated with lower rates of adverse events than those for BQT. Among patients receiving the 14-day VA dual therapy regimen, there was no difference in eradication rates between the high- and low-dose groups. The low-dose 14-day regimen was associated with higher eradication outcomes than the low-dose 10-day regimen. No significant differences in adverse event rates were observed between the various dosage groups.

**Conclusion:**

The 14-day low-dose VA dual regimen offered a favorable balance between efficacy and safety, suggesting that it may be a promising option for *H. pylori* eradication.

**Systematic review registration:**

https://www.crd.york.ac.uk/PROSPERO/view/CRD420261284331, identifier: CRD420261284331.

## 1 Introduction

*Helicobacter pylori* infection affects approximately 43% of the global population (1). In addition to chronic gastritis and peptic ulcers, it is also associated with gastric cancer and gastric MALT lymphoma, as well as extra-gastric diseases such as iron deficiency anemia (2). Current treatment options for *H. pylori* include proton pump inhibitors (PPI)-based Clarithromycin triple therapy, PPI-containing bismuth quadruple therapy (BQT), and potassium-competitive acid blocker (P-CAB)-based regimens. Guidelines recommend BQT as first-line treatment in regions with high clarithromycin resistance rates (≥15%). However, BQT is associated with a higher pill burden and increased incidence of mild adverse events (3).

P-CAB-based treatment regimens, particularly the dual therapy regimen of vonoprazan-amoxicillin, are at least as effective as bismuth-containing quadruple therapy (BQT) and are associated with a lower incidence of adverse events (3). PCABs are a class of acid-suppressing agents that include vonoprazan, tegoprazan, fexuprazan, and keverprazan, and are the most widely used in clinical practice (4). Vonoprazan offers several advantages over conventional PPIs, including rapid onset of action, sustained acid suppression, and efficacy independent of *CYP2C19* genotype or meal timing (5). This potent and rapid acid suppression creates favorable conditions for *H. pylori* eradication by inducing the bacterium to enter a growth-active state, thereby enhancing the bactericidal activity of growth-dependent antibiotics, such as amoxicillin (6). Currently, the optimal duration of vonoprazan–amoxicillin (VA) dual therapy remains unclear, with regimens ranging from 7 to 14 days.

To address this, we performed a network meta-analysis of randomized controlled trials (RCTs) to evaluate the efficacy of various treatment durations, with the aim of providing evidence-based guidance for clinical practice.

## 2 Materials and methods

### 2.1 Literature search strategy

The PubMed, Web of Science, Embase, Cochrane Library, China National Knowledge Infrastructure, Wanfang, VIP Chinese Science and Technology Periodicals Database, and Chinese Medical Association databases were systematically searched for relevant RCTs comparing various durations of VA dual therapy. The literature search covered the period from database inception up to February 1, 2026. The search strategies combined subject headings and free-text terms, including “vonoprazan”, “amoxicillin”, “P-CAB”, and “randomized controlled trials”, using both English and Chinese keywords. This study was registered with the International Prospective Register of Systematic Reviews (i.e., “PROSPERO”) under Accession Number CRD420261284331.

### 2.2 Inclusion criteria

The inclusion criteria were based on the Population, Intervention, Comparator, Outcome, Study Design (i.e., “PICOS”) framework as follows: P, adults with *H. pylori* infection; I, VA dual therapy (7-day, 10-day, or 14-day); C, BQT; O, *H. pylori* eradication rate and incidence of adverse events; S, RCTs.

### 2.3 Exclusion criteria

The exclusion criteria were as follows: (P) non-adult participants; (I) interventions involving dual therapies other than vonoprazan combined with amoxicillin; (C) controls using non-BQT; (O) eradication rates or incidences of adverse events not reported; and (S) study designs were non-RCTs, including reviews, meta-analyses, conference abstracts, dissertations, retrospective studies, ongoing studies, and studies with inaccessible full texts or incomplete data.

### 2.4 Data extraction

All retrieved records were imported into Zotero (www.zotero.org) and duplicates were removed. Two reviewers independently screened the titles and abstracts in accordance with the inclusion and exclusion criteria, and excluded the irrelevant studies. Potentially eligible studies were retrieved for full-text review to determine suitability for final inclusion. Any disagreements between the 2 reviewers were resolved through consensus discussion with a third reviewer.

The following data were extracted from the included studies: first author; publication year; country or region; sample sizes of the intervention and control groups; detailed treatment regimens; methods for *H. pylori* detection; eradication rates based on intention-to-treat (ITT) analysis; and the incidence of adverse events.

### 2.5 Quality assessment of the included studies

Two reviewers independently assessed the quality of the included studies. Disagreements were resolved through discussion or consultation with a third reviewer. The risk of bias (RoB) was evaluated using Cochrane Risk of Bias (RoB) version 2.0 tool, which assesses 5 domains: random sequence generation; deviations from intended interventions; missing outcome data; measurement of the outcome; and selection of the reported result. For each domain, reviewers assigned one of the following judgments: “yes” (Y); “probably yes” (PY); “no” (N); “probably no” (PN); or “not applicable” (NA). Based on these assessments, the overall risk of bias for each study was categorized as “low risk of bias”, “some concerns”, or “high risk of bias” (7).

### 2.6 Statistical analysis

#### 2.6.1 Classification of interventions and node definitions

This study compared VA dual-therapy regimens of varying durations with BQT to identify the optimal treatment duration; simultaneously, it compared high-dose and low-dose VA regimens within different VA treatment protocols to determine the optimal dosage.

The interventions were categorized to facilitate direct and indirect comparisons. For the network meta-analysis, treatments were coded as follows: VA 14-day regimen (VA14 [Group A]); VA 10-day regimen (VA10 [Group B]); VA 7-day regimen (VA7 [Group C]); and BQT [Group D]. For dose-level analysis, regimens were further classified as high-dose VPZ-AMX (HVA) or low-dose VPZ-AMX (LVA).

Several grouping strategies were applied to ensure comparability across studies. First, studies that evaluated both high- and low-dose regimens within the same treatment duration were pooled into a single node (8). Studies with different dosing frequencies but identical total daily doses and treatment durations were combined. A large multicenter randomized controlled trial demonstrated that under the condition of equal total daily doses, different dosing frequencies did not result in statistically significant differences in *H. pylori* eradication rates. Therefore, these studies were pooled for analysis (9). Studies using centrally procured drugs and those using non-centrally procured drugs were consolidated because no statistically significant differences in efficacy were reported between them under the same regimen (10, 11). Finally, subgroup analyses were performed to validate the robustness of the grouping decisions.

#### 2.6.2 Statistical methods

Data analysis was performed using R version 4.5.2 (R Core Team; R Foundation for Statistical Computing, Vienna, Austria). First, a pairwise meta-analysis was performed to compare eradication rates and adverse event incidence between each VA regimen (i.e., VA14, VA10, and VA7) and BQT. All outcomes were dichotomous. Eradication rates were analyzed based on the ITT population. Effect sizes are expressed as odds ratio (*OR*) with corresponding *95%* confidence interval (*CI*). Differences were considered to be statistically significant if the *95% CI* did not include 1. Heterogeneity was assessed using the *I*^2^statistic. Model selection should be based on anticipated clinical heterogeneity rather than solely on statistical thresholds; therefore, a random-effects model was used for the network meta-analysis.

A Bayesian network meta-analysis was performed using the Markov Chain Monte Carlo (MCMC) method in R version 4.5.2. The effect sizes are expressed as *OR* with corresponding *95%* credible interval (*CrI*). Differences were considered to be statistically significant if the *95% CrI* did not include a 1. Network plots were generated for each outcome. In these plots, the size of each node corresponds to the total sample size for that intervention and the thickness of the connecting lines reflects the number of studies providing direct comparisons between interventions. For any closed loops formed in the network, a node-splitting analysis was performed to evaluate inconsistencies by assessing the agreement between direct and indirect evidence using weakly informative priors. Treatment effects and regression coefficients were assigned to normal priors with a mean of 0 and variance of 1,000. The standard deviation of the heterogeneity was assigned a uniform value before (0, 2). Model convergence was assessed using the Gelman–Rubin diagnostic method, which required a potential scale reduction factor of less than 1.05 for all parameters. Convergence was also supplemented by visual inspection of the trace and posterior density plots.

To further determine the optimal dosage and treatment duration of VPZ–AMX dual therapy, pairwise and network meta-analyses were performed at the dosage level for the VA14 and VA10 regimens, respectively. The outcome measures and effect sizes were consistent with those in the main analyses. All network plots were generated using R version 4.5.2.

Publication bias was assessed by visually inspecting funnel plots constructed using Stata Release 18.0 (Stata Corp LLC, College Station, TX, USA).

## 3 Results

### 3.1 Literature search and screening results, and general characteristics

**Figure 1:**
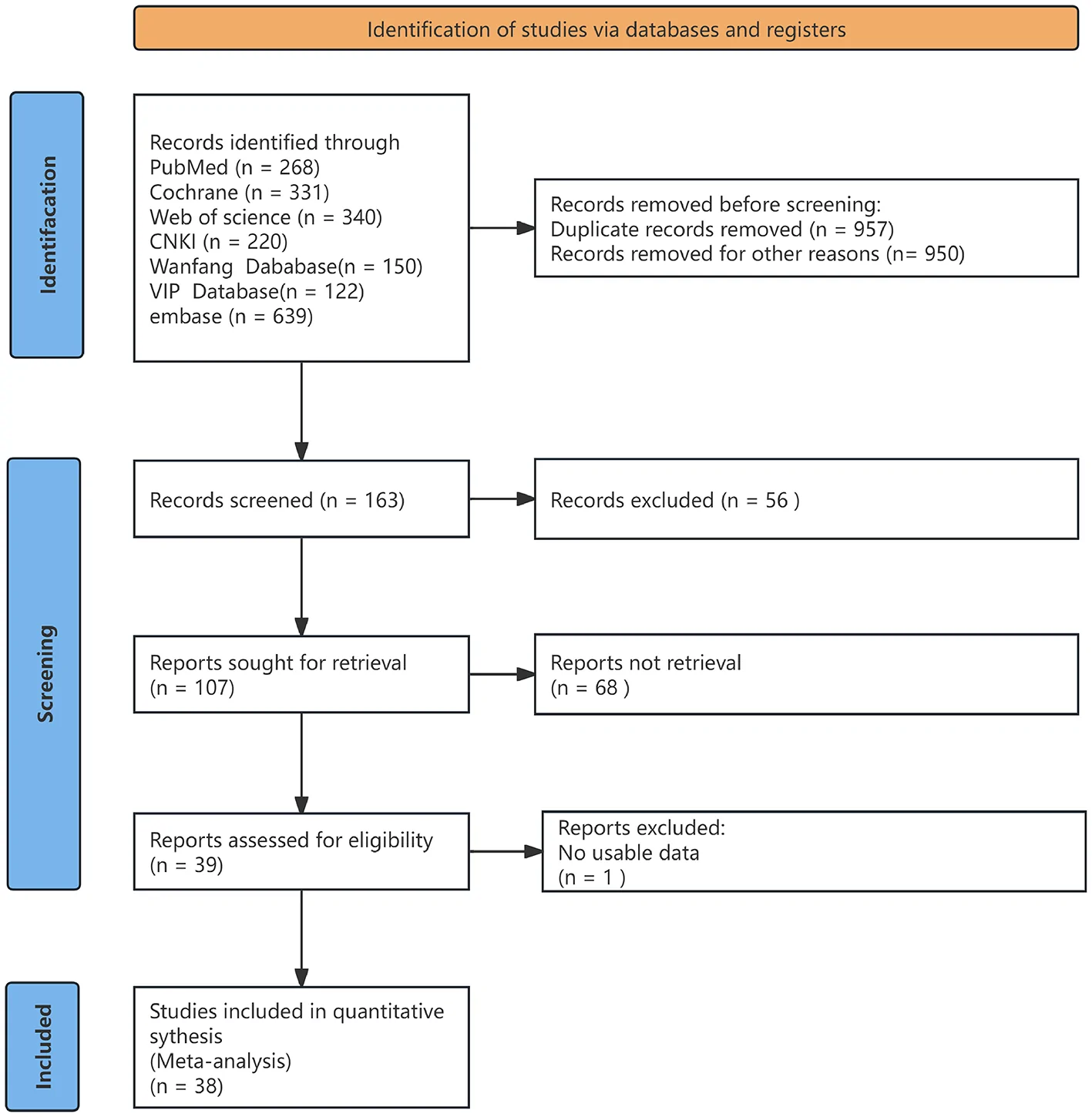
Flow diagram of literature screening.

The initial literature search retrieved 2,201 studies, of which 38 were ultimately included in the analysis. A flow-diagram illustrating the study-selection process and results is presented in Figure 1. The 38 included RCTs comprised 10,489 participants and were published between 2022 and 2025 (12–49). The characteristics of the included studies are summarized in Table 1.

**Table 1:** Characteristics of included studies.

Reference	Year	Country	Number of patients	Eradication regimen				
				Dose	Experimental group	Control group	Eradication rate (ITT)	Adverse events
Yang et al. (12)	2023	China	VA14: 200 VA10: 200 BQT: 200	HVA14 HVA10	VA14: VPZ 20 mg BID + AMO 1,000 mg TID VA10: VPZ 20 mg BID + AMO 1,000 mg TID	BQT: RBZ 20 mg BID + Bismuth/Tnz/CLA combination 4,200 mg BID	VA14: 86.0% (172/200) VA10: 87.0% (174/200) BQT:70.5% (141/200)	VA14: 9.5% (17/200) VA10: 8.5% (15/200) BQT: 17% (32/200)
Yan et al. (13)	2024	China	VA14: 142 VA10: 142	HVA14 HVA10	VA14: VPZ 20 mg BID + AMO 750 mg QID	VA10: VPZ 20 mg BID + AMO 1,000 mg TID	VA14: 90.1% (128/142) VA10: 88.7% (126/142)	VA14: 12.7% (18/142) VA10: 5.6% (8/142)
Fan et al. (14)	2025	China	VA14: 252 BQT: 252		VA14: VPZ 20 mg BID + AMO 1,000 mg BID	BQT: ESO 20 mg BID + Bismuth 220 mg BID + AMO 1,000 mg BID + CLA 500 mg BID	VA14: 79.4% (200/252) BQT: 85.7% (216/252)	VA14: 27.2% (67/246) BQT: 42.7% (105/246)
Dong et al. (15)	2024	China	VA10: 455 VA14: 459		VA10: VPZ 20 mg BID + AMO 1,000 mg TID	VA14: VPZ 20 mg BID + AMO 1,000 mg TID	VA10: 88.79% (404/455) VA14: 92.37% (424/459)	VA10: 20.0% (91/455) VA14: 17.9% (82/459)
Peng et al. (16)	2024	China	HVA10: 172 LVA10: 172 HVA14: 172	HVA14 HVA10 LVA10	HVA10: VPZ 20 mg BID + AMO 750 mg QID LVA10: VPZ 20 mg BID + AMO 1,000 mg BID	HVA14: VPZ 20 mg BID + AMO 750 mg QID	HVA10: 86.6% (149/172) LVA10: 79.7% (137/172) HVA14: 89.5% (154/172)	HVA10:19.8% (34/174) LVA10: 17.4% (30/172) HVA14: 22.7% (39/172)
Jiang et al. (17)	2024	China	VA14: 200 BQT: 200		VA14: VPZ 20 mg BID + AMO 1,000 mg TID	BQT: RBZ 10 mg BID + AMO 1,000 mg BID + CLA 500 mg BID + Bismuth 600 mg BID	VA14: 94.0% (188/200) BQT: 87.0% (174/200)	VA14: 19.0% (38/200) BQT: 53.0% (106/200)
Hu et al. (18)	2022	China	LVA10: 37 HVA10: 37 LVA7: 24 HVA7: 21		LVA10: VPZ 20 mg BID + AMO 1,000 mg BID HVA10: VPZ 20 mg BID + AMO 1,000 mg TID	LVA7: VPZ 20 mg BID + AMO 1,000 mg BID HVA7: VPZ 20 mg BID +AMO 1,000 mg TID	LVA10: 89.2% (33/37) HVA10: 81.1% (30/37) LVA7: 66.7% (16/24) HVA7: 81.0% (17/21)	LVA10:29.7% (11/37) HVA10:24.3% (9/37) LVA7:16.7% (4/24) HVA7:23.8% (5/21) Tao et al. (19)
Tao et al. (19)	2025	China	VA-T7: 125 VA-Q7: 125 VA-T10: 125 VA-Q10: 125	HVA7 HVA10	VA-T7: VPZ 20 mg BID + AMO 1,000 mg TID VA-Q7: VPZ 20 mg BID + AMO 750 mg QID	VA-T10: VPZ 20 mg BID + AMO 1,000 mg TID VA-Q10: VPZ 20 mg BID + AMO 750 mg QID	VA-T7: 84.0% (105/125) VA-Q7: 81.6% (102/125) VA-T10: 91.2% (114/125) VA-Q10: 90.4% (113/125)	VA-T7: 11.2% (14/125) VA-Q7: 10.4% (12/125) VA-T10:12.8%(16/125) VA-Q10:16.0%(20/125)
Cheng et al. (20)	2026	China	VA10: 150 BQT: 150 BQT (NCDP): 150		VA10: VPZ 20 mg BID + AMO 750 mg QID	BQT: ESO 20 mg BID + Bismuth 200 mg BID + AMO 1,000 mg BID + CLA 500 mg BID BQT (NCDP): ESO 20 mg BID +Bismuth 200 mg BID +AMO (NCDP) 1,000 mg BID + CLA 500 mg BID	VA10: 88.0% (132/150) BQT: 85.3% (128/150) BQT (NCDP): 84.6% (127/150)	VA10:6.8% (10/147) BQT:17.9% (26/145) BQT (NCDP): 19.0% (28/147)
Song et al. (21)	2025	China	VA10: 209 VA14: 209	HVA14 HVA10	VA10: VPZ 20 mg BID + AMO 1,000 mg TID	VA14: VPZ 20 mg BID + AMO 1,000 mg TID	VA10: 83.3% (174/209) VA14: 88.0% (184/209)	VA10: 15.7% (32/204) VA14: 17.6% (36/204)
Lin et al. (22)	2024	China	VA10: 125 VA14: 125	HVA14 HVA10	VA10: VPZ 20 mg BID + AMO 1,000 mg TID	VA14: VPZ 20 mg BID + AMO 1,000 mg TID	VA10: 89.60% (112/125) VA14: 91.20% (114/125)	VA10: 31.7% (39/123) VA14: 36.1% (44/122)
Qian et al. (23)	2023	China	HVA10: 125 VA10: 125 BQT: 125	HVA10 LVA10	HVA10: VPZ 20 mg BID + AMO 750 mg QID VA10: VPZ 20 mg BID + AMO 1,000 mg BID	BQT: ESO 20 mg BID + Bismuth 200 mg BID + AMO 1,000 mg BID + CLA 500 mg BID	HVA10: 91.2% (114/125) VA10: 82.4% (103/125) BQT: 88.0% (110/125)	HVA10: 11.4% (14/123) VA10: 8.2% (10/122) BQT: 23.58% (29/123)
Yan et al. (24)	2024	China	VA10: 157 BQT: 157		VA10: VPZ 20 mg BID + AMO 1,000 mg TID	BQT: RBZ 10 mg BID + AMO 1,000 mg BID + CLA 500 mg BID + Bismuth 200 mg BID	VA10: 86.0%% (135/157) BQT: 89.2% (140/157)	VA10: 21.0% (33/157) BQT: 43.9% (69/157)
Huang et al. (25)	2024	China	VA10: 75 BQT: 73		VA10: VPZ 20 mg BID + AMO 1,000 mg TID	BQT: ILA 10 mg BID + Bismuth 220 mg BID + AMO 1,000 mg BID + CLA 500 mg BID	VA10: 93.2% (96/103) BQT: 80.2% (81/101)	VA10: 25.3% (25/75) BQT: 37.6% (38/733)
Qi et al. (26)	2025	China	VA14: 85 BQT: 85		VA14: VPZ 20 mg BID + AMO 1,000 mg TID	BQT: RBZ 20 mg BID + Bismuth 220 mg BID + AMO 1,000 mg BID + FZD 100 mg BID	VA14: 91.8% (78/85) BQT: 88.2% (75/85)	VA14: 6.1% (5/82) BQT14: 45.8% (38/83)
Peng et al. (27)	2023	China	VA14: 158 BQT: 158		VA14: VPZ 20 mg BID + AMO 750 mg QID	BQT: ESO 20 mg BID + AMO 1,000 mg BID + CLA 500 mg BID + Bismuth 220 mg BID	VA14: 89.9% (142/158) BQT: 81.0% (128/158)	VA14: 19.0% (30/158) BQT: 43.0% (68/158)
Zhang et al. (28)	2025	China	VA14: 125 BQT: 125		VA14: VPZ 20 mg BID + AMO 1,000 mg TID	BQT: LAN 30 mg BID + CLA 500 mg BID + AMO 1,000 mg BID + Bismuth 240 mg BID	VA14: 92.0% (115/125) BQT: 88.0% (110/125)	VA14: 11.2% (14/125) BQT: 20.8% (26/125)
Li et al. (29)	2023	China	VA14: 75 BQT: 75		VA14: VPZ 20 mg BID + AMO 750 mg TID	BQT: ESO 20 mg BID + AMO 1,000 mg BID + FZD 100 mg BID + Bismuth 220 mg BID	VA14: 77.33% (58/75) BQT: 78.67% (59/75)	VA14: 9.38% (6/64) BQT: 22.95% (14/61)
Chen et al. (30)	2024	China	VA14: 45 BQT: 45		VA14: VPZ 20 mg BID + AMO 1,000 mg TID	BQT: ILA 5 mg BID + FZD 100 mg BID + Bismuth 240 mg BID	VA14: 84.4% (38/45) BQT: 84.4% (38/45)	VA14: 2.3% (1/43) BQT: 14.0% (6/43)
Cheung et al. (31)	2024	China	VA14: 58 BQT: 57		VA14: VPZ 20 mg BID + AMO 750 mg TID	BQT: OME 40 mg qd + AMO 1,000 mg BID + FZD 100 mg BID + Bismuth 150 mg TID	VA14: 84.5% (49/58) BQT: 84.2% (48/57)	VA14: 7.4% (4/54) BQT: 18.9% (10/53)
Hu et al. (32)	2023	China	VA14: 56 BQT: 44		VA14: VPZ 20 mg BID + AMO 1,000 mg BID	BQT: RBZ 10 mg BID + Bismuth 200 mg TID + CLA 500 mg BID + AMO 1,000 mg BID	VA14: 94.64% (53/56) BQT: 81.82% (36/44)	VA14: 7.14% (4/56) BQT: 22.73% (10/44)
Wang et al. (33)	2023	China	VA14: 96 BQT: 93		VA14: VPZ 20 mg BID + AMO 1,000 mg TID	BQT: OME 20 mg BID + Bismuth 220 mg BID + AMO 1,000 mg BID + CLA 500 mg BID	VA14: 91.0% (91/96) BQT: 79.0% (79/93)	VA14:4.2% (4/96) BQT: 5.4% (5/93)
Duan et al. (34)	2023	China	VA7: 60 BQT: 60		VA7: VPZ 20 mg BID + AMO 500 mg TID	BQT: OME 20 mg BID + Bismuth 110 mg TID + AMO 1,000 mg BID + CLA 500 mg BID	VA7: 88.3% (53/60) BQT: 73.3% (44/60)	VA7: 23.7% (14/59) BQT: 49.2% (29/59)
Zhu et al. (35)	2024	China	VA14: 100 BQT: 100		VA14: VPZ 20 mg BID + AMO 1,000 mg TID	BQT: RBZ 20 mg BID + AMO 1,000 mg BID + CLA 500 mg BID + Bismuth 200 mg BID	VA14: 90.0% (90/100) BQT: 79.0% (79/100)	VA14: 11.6% (11/95) BQT: 14.6% (14/96)
Liu et al. (36)	2024	China	VA14: 60 BQT: 60		VA14: VPZ 20 mg BID + AMO 1,000 mg TID	BQT: OME 20 mg BID + Bismuth 200 mg BID + AMO 1,000 mg BID + CLA 500 mg BID	VA14: 95.0% (57/60) BQT: 83.3% (50/60)	VA14: 8.33% (5/60) BQT: 23.33% (14/60)
Wang et al. (37)	2023	China	VA14: 106 BQT: 104		VA14: VPZ 20 mg BID + AMO 1,000 mg TID	BQT: RBZ 20 mg BID + Bismuth 600 mg BID + CLA 500 mg BID + AMO 1,000 mg BID	VA14: 89.6% (95/106) BQT: 92.3% (96/104)	VA14: 4.7% (5/106) BQT: 20.2% (21/104)
Xiao et al. (38)	2025	China	VA14: 100 BQT: 100		VA14: VPZ 20 mg BID + AMO 1,000 mg TID	BQT: Bismuth 220 mg BID + ESO 20 mg BID + TET 500 mg TID + MTZ 500 mg QID	VA14: 96.0% (96/100) BQT: 92.0% (92/100)	VA14: 39.0% (39/100) BQT: 71.0% (71/100)
Xie et al. (39)	2025	China	VA14: 97 BQT: 97		VA14: VPZ 20 mg BID + AMO 1,000 mg TID	BQT: ESO 20 mg BID + Bismuth 220 mg BID + AMO 1,000 mg BID + MTZ 400 mg QID	VA14: 88.7% (86/97) BQT: 91.8% (89/97)	VA14: 16.67% (15/90) BQT: 38.04% (35/92)
Yang et al. (40)	2024	China	VA10: 75 VA14: 75	LVA14 LVA10	VA10: VPZ 20 mg BID + AMO 750 mg TID	VA14: VPZ 20 mg BID + AMO 750 mg TID	VA10: 86.7% (65/75) VA14: 90.7% (68/75)	VA10: 12.9% (9/70) VA14: 9.7% (7/72)
Jiang et al. (41)	2024	China	VA14: 100 BQT: 100		VA14: VPZ 20 mg BID + AMO 1,000 mg TID	BQT: OME 20 mg BID + MTZ 400 mg QID + TET 500 mg TID + Bismuth 600 mg BID	VA14: 96. 0% (96/100) BQT: 92.0% (92/100)	VA14: 39.0% (39/100) BQT: 71.0% (71/100)
Deng et al. (42)	2024	China	VA10: 75 BQT: 75		VA10: VPZ 20mg BID + AMO 1,000 mg BID	BQT: OME 20 mg BID + AMO 1,000 mg BID + MTZ 400 mg TID + Bismuth 200 mg TID	VA10: 85.3% (64/75) BQT: 76.0% (57/75)	VA10: 4.29% (3/70) BQT: 14.71% (10/68)
Shang et al. (43)	2024	China	VA7: 80 VA14: 80 BQT: 80		VA7: VPZ 20 mg BID + AMO 1,000 mg BID VA14: VPZ 20 mg BID + AMO 1,000 mg BID	BQT: RBZ 20 mg BID + Bismuth 200 mg BID + AMO 1,000 mg BID + CLA 500 mg BID	VA7: 76.3% (61/80) VA14: 83.8% (67/80) BQT: 80.0% (64/80)	VA7: 8.8% (7/80) VA14: 11.3% (9/80) BQT: 8.8% (7/80)
Jin et al. (44)	2024	China	VA7: 63 BQT: 63		VA7: VPZ 20 mg BID + AMO 500 mg TID	BQT: OME 20 mg BID + AMO 1,000 mg BID + CLA 500 mg BID + Bismuth 150 mg QID	VA7: 95.2% (60/63) BQT: 84.1% (53/63)	VA7: 2.3% (1/63) BQT: 11.6% (5/63)
Chen et al. (45)	2022	China	VA7: 50 BQT: 50		VA7: VPZ 20 mg BID + AMO 750 mg QID	BQT: OME 20 mg BID + AMO 1,000 mg BID + CLA 500 mg BID + Bismuth 220 mg BID	VA7: 94% (47/50) BQT: 88% (44/50)	VA7: 12% (6/50) BQT: 54% (17/50)
Jian and He (46)	2023	China	VA14: 74 BQT: 77		VA14: VPZ 20 mg BID + AMO 750 mg QID	BQT: RBZ 10 mg BID + Bismuth 220 mg BID + AMO 1,000 mg BID + CLA 500 mg BID	VA14: 94.6% (70/74) BQT: 87% (67/77)	VA14: 39.2% (29/74) BQT: 79.2% (61/77)
Liang et al. (47)	2025	China	VA14: 120 BQT: 120		VA14: VPZ 20 mg BID + AMO 1,000 mg TID	BQT: Bismuth 220 mg BID + RBZ 20 mg BID + AMO 1,000 mg BID + FZD 100 mg BID	VA14: 89.2% (107/120) BQT: 87.5% (105/120)	VA14: 6.9% (8/116) BQT: 21.7% (25/115)
Chen et al. (48)	2025	China	VA14: 150 BQT: 150		VA14: VPZ 20 mg BID +AMO 1,000 mg TID	BQT: Bismuth 220 mg BID + AMO 1,000 mg BID + CLA 500 mg BID + OME 20 mg BID	VA14: 91.3% (137/150) BQT: 82.6% (124/150)	VA14: 6.76% (10/148) BQT: 23.57% (33/140)
Guo et al. (49)	2024	China	VA14: 280 BQT: 280		VA14: VPZ 20 mg BID + AMO 1,000 mg BID	BQT: LAN 30 mg BID + AMO 1,000 mg BID + CLA 500 mg BID + Bismuth 600 mg BID	VA14: 88.6% (248/280) BQT: 84.3% (236/280)	VA14: 7.45% (20/264) BQT: 12.1% (32/268)

AMO, amoxicillin; VPZ, vonoprazan; BID, twice per day; TID, three times per day; QID, four times per day; HVA, high dose vonoprazan and amoxicillin; LVA, low dose vonoprazan and amoxicillin; VA14, vonoprazan-amoxicillin 14-day regimen; VA10, vonoprazan-amoxicillin 10-day regimen; VA7, vonoprazan-amoxicillin 7-day regimen; BQT, PPI-containing Bismuth quadruple therapy; ITT, intention-to-treat; RBZ, rabeprazole; TNZ, tinidazole; CLA, Clarithromycin; Bismuth, bismuth-containing agent; ESO, Esomeprazole; ILA, Ilaprazole; FZD, furazolidone; LAN, Lansoprazole; OME: Omeprazole; TET, Tetracycline; MTZ, metronidazole; NCDP, national centralized drug procurement.

The methodological quality of the included studies was assessed using the Cochrane RoB 2.0 tool. Overall, the 38 included studies demonstrated a low risk of bias. The results of the quality assessment are reported in Supplementary Figure 1.

### 3.2 Comparison of various VA regimens and with BQT

#### 3.2.1 Eradication rates and adverse events for VA vs. BQT

**Figure 2:**
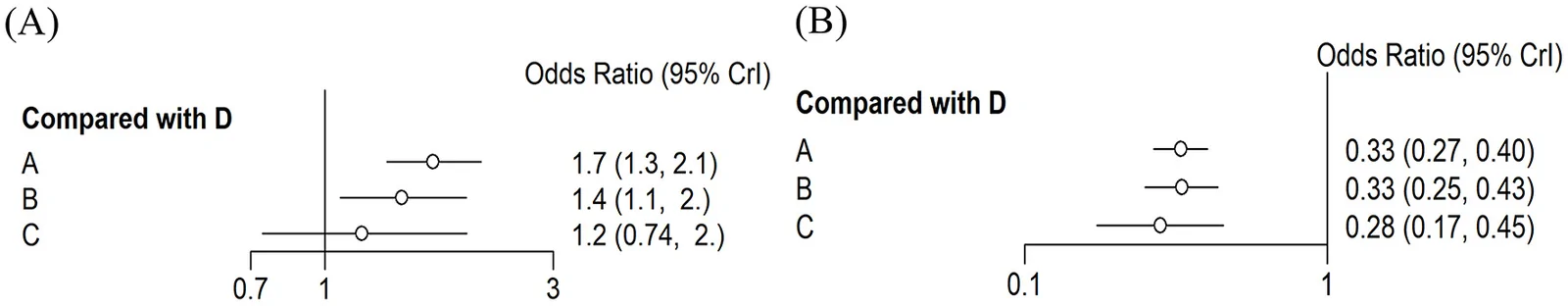
The comparison of VA versus BQT in *Helicobacter pylori* eradication rates and adverse event rates. Compared to Group D, Group A [*OR* = 1.7, *95% CrI* (1.4, 2.1)] and Group B [*OR* = 1.4, *95% CrI* (1.1, 2.0)] demonstrated significantly higher eradication rates **(A)**. Compared to Group D, Group A [*OR* = 0.33, *95% CrI* (0.27, 0.40)], Group B [*OR* = 0.33, *95% CrI* (0.25, 0.43)], and Group C [*OR* = 0.28, *95% CrI* (0.17, 0.45)] **(B)**.

The network meta-analysis, incorporating both direct and indirect evidence, revealed that compared with Group D, Groups A and B achieved significantly higher eradication rates, with the differences being statistically significant. No significant differences were observed between groups C and D. The Surface Under the Cumulative Ranking (i.e., “SUCRA”) indicated that VA14 was the most effective regimen, whereas BQT was the least effective (Figure 2A and Supplementary Figure 1, Supplementary Tables 1, 3). All three dual-therapy regimens were associated with significantly lower adverse event rates than those in Group D (Figure 2B and Supplementary Table 2). The between-study heterogeneity variance (τ^2^) was 0.15 (*95% CrI* 0.05–0.36) for eradication rates and 0.09 (*95% CrI* 0.01–0.25) for adverse events.

### 3.3 Comparison of high- and low-dose regimens of various VA regimens

#### 3.3.1 Eradication rates and adverse events for high-dose VA14 versus high-dose VA10

Across the included studies, the vonoprazan dose was consistent, whereas the amoxicillin doses varied. Therefore, studies were stratified based on a daily amoxicillin dose of 3,000 mg, categorizing them into high-dose (HVA; ≥3,000 mg/day) and low-dose (LVA; < 3,000 mg/day) strata. Then, the efficacy and safety of different VA regimens were compared across groups.

**Figure 3:**
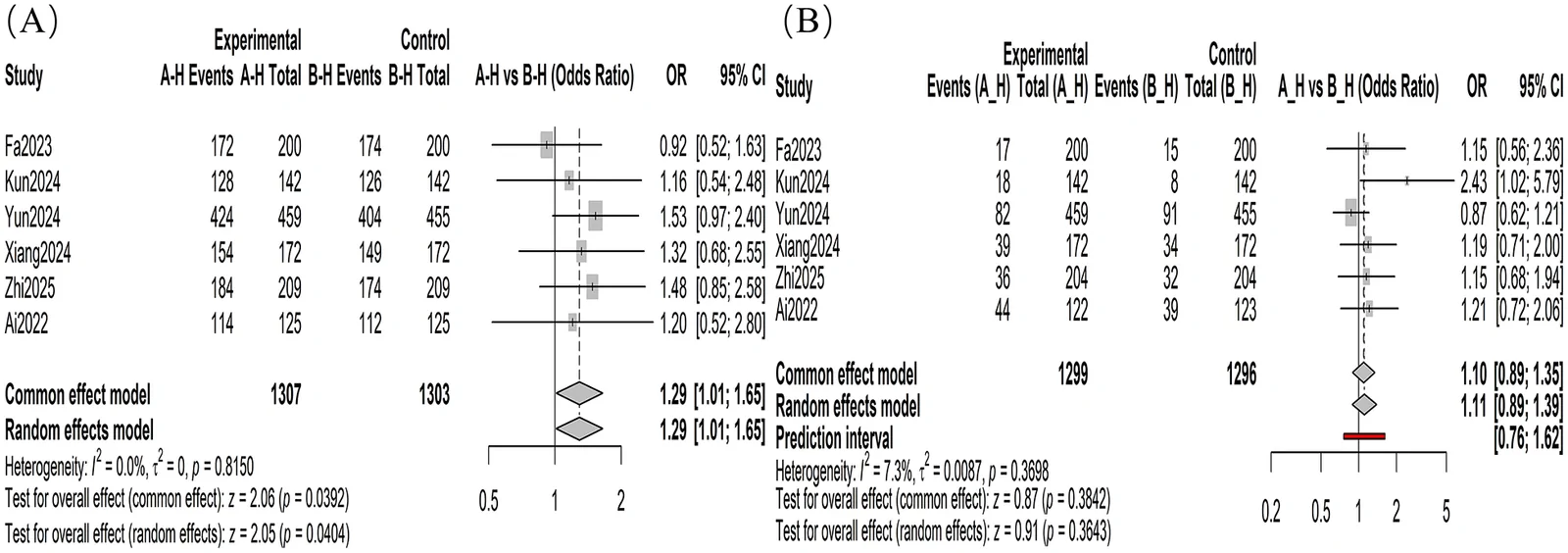
The comparison of high-dose VA 14 vs. VA10 on *Helicobacter pylori* eradication rates and adverse event rates. In the high-dose stratum, Group A demonstrated significantly higher eradication rates than Group B (*OR* = 1.29, *95% CI* [1.01, 1.65], *P* < 0.05) **(A)**. No statistically significant differences were observed in the high-dose stratum (*P* > 0.05) **(B)**.

Meta-analysis revealed that, in the high-dose stratum, the eradication rate of Group A was significantly higher than that of Group B, and this difference was statistically significant (Figure 3A).

In the high-dose stratum, there was no statistically significant difference in the incidence of adverse events between Group A and Group B (*P* > 0.05; Figure 3B).

#### 3.3.2 Eradication rates and adverse events for various VA regimens

**Figure 4:**
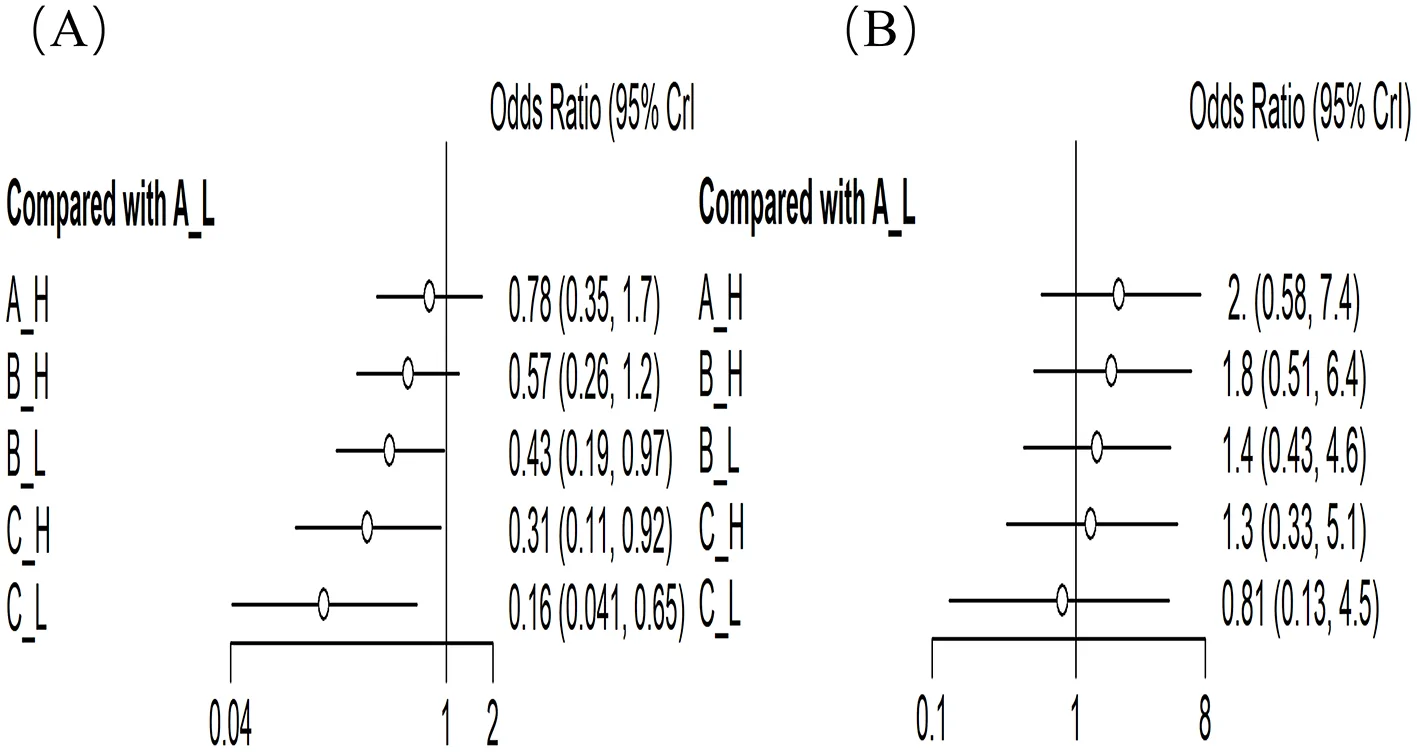
The comparison of various VA regimens in *Helicobacter pylori* eradication rates and adverse event rates. Low-dose Group A demonstrated significantly higher eradication rates than low-dose Group B [*OR* = 0.43, *95% CrI* (0.19, 0.97)], as well as the high- and low-dose regimens of Group C (*P* < 0.05) **(A)**. No statistically significant differences were observed within the different VA dosage groups (all *P* > 0.05) **(B)**.

The network meta-analysis revealed no statistically significant differences in eradication rates between high-dose and low-dose regimens within Group A. However, the low-dose Group A demonstrated significantly higher eradication rates than the low-dose Group B, and the difference was statistically significant (Figure 4A).

Regarding the incidence of adverse events, there were no statistically significant differences between high- and low-dose regimens across different VA regimens (Figure 4B).

#### 3.3.3 Adherence comparisons across regimens

The adherence analysis was restricted to studies reporting good adherence, which was defined as patients consuming at least 80% of the study medication. This network meta-analysis included 21 studies that reported compliance data. The results showed a statistically significant difference in adherence between Group B and Group D (*P* < 0.05), In contrast, no statistically significant difference in adherence was observed among the VA regimens themselves (All *P* > 0.05; Supplementary Figure 3).

### 3.4 Network meta regression

The year of publication was included as a covariate in a network meta-regression model. The results showed no significant modification of treatment effects by study year, with a regression coefficient of −0.09 (*95% CrI* −0.59 to 0.40), indicating no evidence that treatment effects varied across years (*P* > 0.05).

### 3.5 Model diagnostics and sensitivity analyses

#### 3.5.1 Network plots

**Figure 5:**
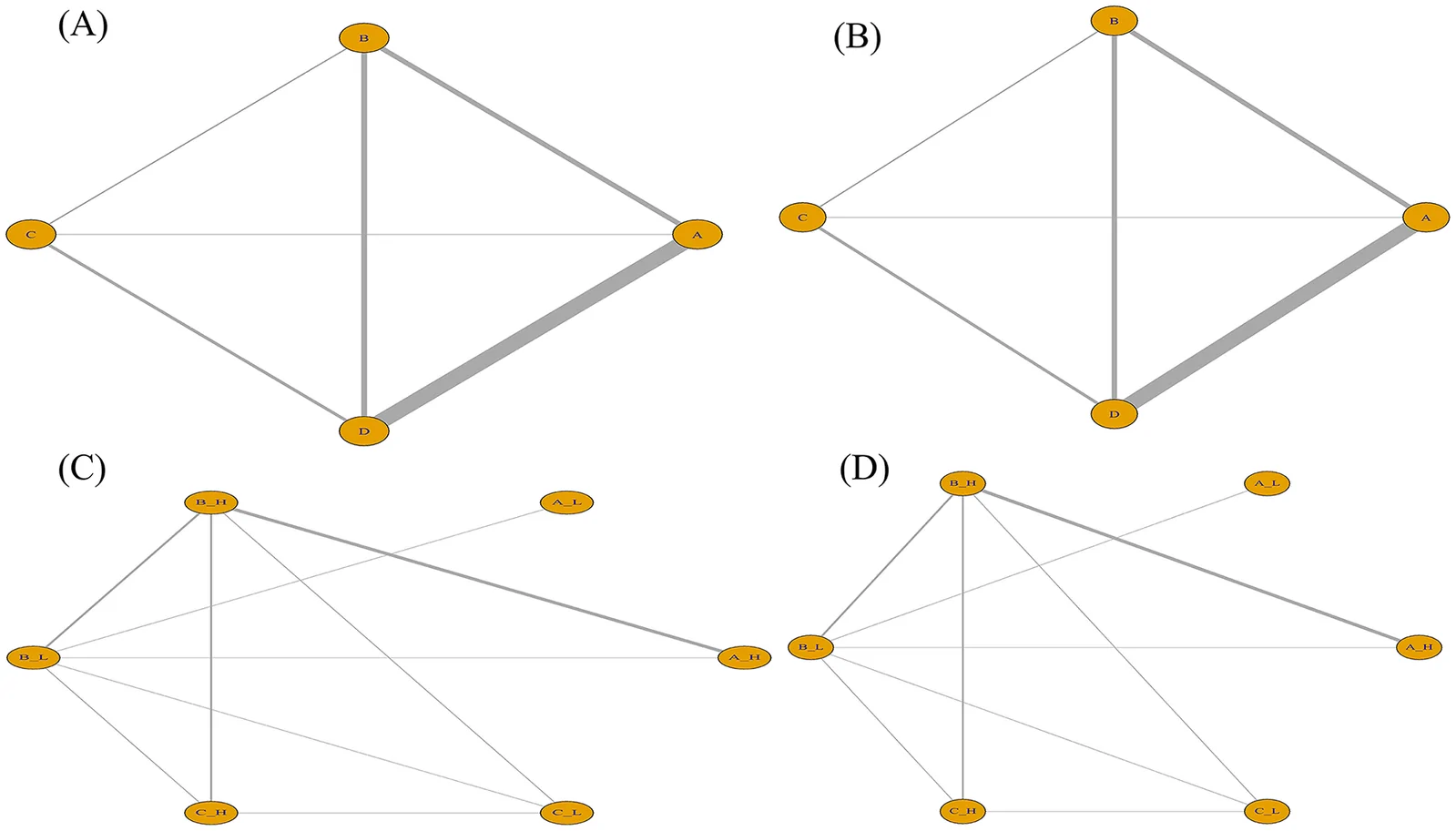
Network geometry comparing different vonoprazan-amoxicillin (VA) regimens versus bismuth-containing quadruple therapy (BQT) for *Helicobacter pylori* eradication rates **(A)** and adverse event rates **(B)**, and high-dose versus low-dose of different VA regimens for *Helicobacter pylori* eradication rates **(C)** and adverse event rates **(D)**. Node A stands for 14-day VA regimen; Node B stands for 10-day VA regimen; Node C stands for 7-day VA regimen; Node D stands for BQT; Node A_H stands for high-dose 14-day VA; Node A_L stands for low-dose 14-day VA; Node B_H stands for high-dose 10-day VA; Node B_L stands for low-dose 10-day VA; Node C_H stands for high-dose 7-day VA; Node C_L stands for low-dose 7-day VA.

The network geometry for comparisons of different VA regimens vs. BQT, as well as high-dose vs. low-dose of different VA regimens, is presented for eradication rates and adverse event incidence, is presented in Figure 5.

#### 3.5.2 Inconsistency

**Figure 6:**
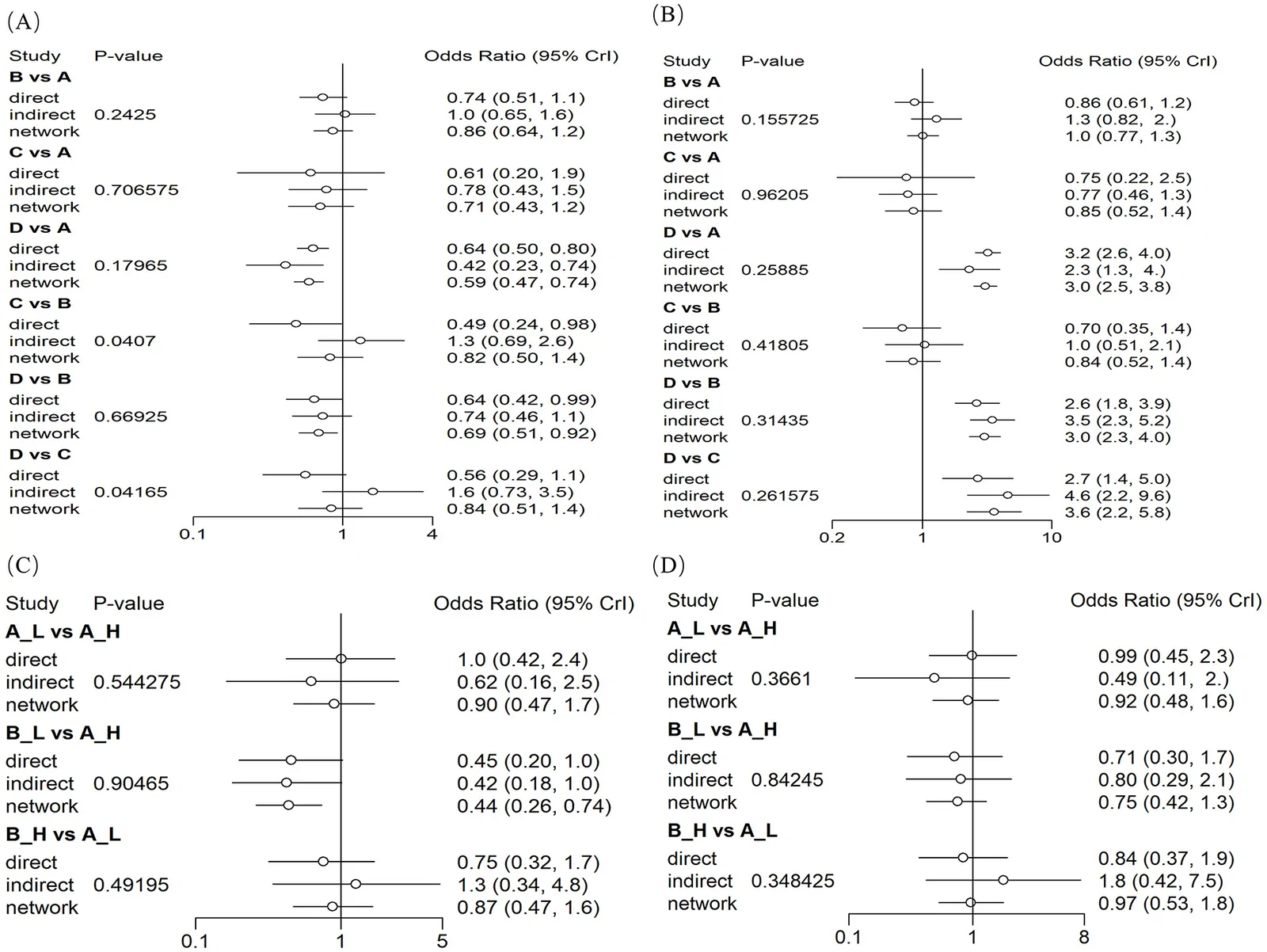
Inconsistency assessment in the comparison of VA vs. BQT and of different doses of VA regimens for *Helicobacter pylori* eradication rates and adverse event rates. Significant inconsistencies were detected in the eradication rates between groups B and C (*P* = 0.040) and between groups D and C (*P* = 0.041) **(A, B)**. In contrast, for comparisons between different doses of VA regimens, no significant inconsistency was detected in eradication rates (*P* > 0.05). Safety outcomes demonstrated good consistency across all treatment comparisons, with no statistically significant differences between direct and indirect evidence (*P* > 0.05) **(C, D)**.

Inconsistency was assessed using node-splitting analysis for closed-loop networks. Significant inconsistencies were detected in the eradication rates between groups B and C (*P* = 0.040) and between groups D and C (*P* = 0.041; Figures 6A, B). In contrast, for comparisons between different doses of VA regimens, no significant inconsistency was detected in eradication rates (*P* > 0.05). Safety outcomes demonstrated good consistency across all treatment comparisons, with no statistically significant differences between direct and indirect evidence (*P* > 0.05). The estimates from the direct, indirect, and network meta-analyses were highly consistent, indicating that the findings in this domain were robust and reliable (Figures 6C, D).

Notably, two head-to-head RCTs directly compared Group B with Group C. Pooled analysis of these studies demonstrated that Group B achieved significantly higher eradication rates than Group C (*OR* = 2.06, *95% CI* 1.29–3.28; *P* < 0.05), with no heterogeneity observed between studies (*P* > 0.05; Supplementary Figure 4).

#### 3.5.3 Risk of bias assessment

Regarding publication bias, inspection of the comparison-adjusted funnel plots revealed no significant asymmetry for either eradication rates or adverse event incidence across comparisons of different VA durations vs. BQT as well as for high-dose vs. low-dose comparisons of different VA regimens. The distribution of study points was generally symmetrical, further supporting the robustness of the results (Supplementary Figure 5).

#### 3.5.4 MCMC convergence diagnostics

Convergence of the Markov chains was assessed by calculating the Gelman–Rubin shrinkage factor (R-hat) and visually inspecting the trace plots. The results showed that all parameters had R-hat values below 1.05 (maximum 1.02), and the trace plots indicated good mixing with no obvious trends or drifts, demonstrating adequate convergence and reliable posterior estimates (Supplementary Figures 6, 7).

#### 3.5.5 Sensitivity analysis

Node-splitting analysis revealed Significant inconsistencies were detected in the eradication rates between groups B and C (*P* = 0.040), and between groups D and C (*P* = 0.041). To explore the source of these inconsistencies, a leave-one-out sensitivity analysis was performed by sequentially excluding each study and rerunning node splitting. The results showed that after excluding only Jian 2024 (34), the *P*-values for groups B and C (*P* = 0.055) and groups D and C (*P* = 0.058) were both greater than 0.05, and the inconsistencies were no longer statistically significant. When other studies were excluded, the inconsistencies persisted. This indicated that the observed inconsistency originated from a single study (Supplementary Figure 8).

To assess the robustness of the results, a sensitivity analysis was performed by replacing the random effects model with a fixed-effects model and rerunning the network meta-analysis. Under the fixed-effect model, the difference in eradication rates between VA14 and BQT was statistically significant (*OR* = 1.6, *95% CrI* 1.4–1.9), as was the difference between VA10 and BQT (*OR* = 1.4, *95% CrI* 1.1–1.7); the difference between VA7 and BQT remained non-significant (*OR* = 1.1, *95% CrI* 0.74–1.5). These results are consistent with the direction and significance of the effect estimates obtained from the random-effects model, indicating that the conclusions of this study do not depend on the choice of the effect model and are robust (Supplementary Figure 9).

Prior sensitivity showed that after using a wider heterogeneity prior of Uniform (0, 5), the effect sizes of all key comparisons and the treatment rankings remained consistent with those of the primary analysis, confirming that the conclusions are not influenced by the choice of priors.

## 4 Discussion

The Maastricht VI/Florence Consensus Report explicitly states that PCAB-based regimens outperform traditional PPI-based triple therapy as both first- and second-line treatments for *H. pylori* infection (50). The present network meta-analysis compared eradication rates and safety profiles of VA dual therapy vs. BQT, and further evaluated the efficacy and safety of various amoxicillin dosages within vonoprazan-based dual therapy. The main findings were as follows. VA14 and VA10 yielded superior eradication rates and demonstrated better safety profiles than BQT, whereas VA7 exhibited only a safety advantage over BQT, without a significant difference in eradication efficacy. Within the dual-therapy regimens, low-dose VA14 achieved significantly higher eradication rates than low-dose VA10 while demonstrating efficacy comparable with high-dose VA14. Five RCTs confirmed the non-inferiority of low-dose VA14 over the high-dose regimen (9, 51–54).

Recent studies have reported that a low-dose VA7 regimen can achieve acceptable eradication rates (55, 56). However, in the present network meta-analysis, VA7 demonstrated significantly lower eradication rates than VA14 and VA10, consistent with 2 Chinese RCTs: one was terminated early for failing the interim efficacy criteria (25); and the other reported unsatisfactory eradication (18). These findings suggest that extending the treatment duration is necessary for the Chinese population, indicating that duration is a key efficacy determinant. A possible explanation for the lower efficacy of the VA7 dual therapy in China than in Japan is the difference in body size between the 2 populations because previous studies have reported that both body weight and body surface area significantly influence treatment success (41, 57). The antibacterial activity of amoxicillin depends on the duration for which its blood concentration remains above its minimum inhibitory concentration (MIC). At a given dose, a larger body size results in lower blood drug levels and a shorter *T* > MIC. In addition, the MIC_90_ for amoxicillin for *H. pylori* in China is 4 mg/L in China (58), whereas it is generally lower 0.06 mg/L in Japan (59). Consequently, at the same amoxicillin dose, Chinese patients achieved lower blood drug concentrations and a shorter effective kill time, making it difficult to achieve a satisfactory eradication rate using the 7-day regimen in China.

Network meta-analysis revealed that the eradication rate for low-dose VA10 was lower than that for both high- and low-dose VA14 and high-dose VA10. Furthermore, one study revealed that the high-dose VA10 regimen had a lower eradication rate than BQT, primarily due to poor patient compliance in the high-dose group, which led to treatment failure. This suggests that compliance may be a significant factor influencing eradication rates (24).

In this network meta-analysis, VA10 showed significantly better adherence than the BQT regimens, whereas no significant difference in adherence was observed among the VA regimens themselves. Given that adherence is an important determinant of eradication success, the superior eradication rates of VA10 over BQT regimens may be partly attributable to its higher adherence. Node-splitting analysis revealed significant inconsistencies in the comparisons between VA10 and VA7, and between VA7 and BQT. A leave-one-out sensitivity analysis indicated that these inconsistencies were driven by a single small-sample study; after excluding this study, the network became consistent. This finding suggests that the observed inconsistency is likely due to imprecise direct evidence from a small sample rather than a systematic bias. Furthermore, caution is warranted when interpreting the treatment rankings based on SUCRA and P score in this study. SUCRA and P score are probabilistic ranking measures that reflect the probability of a treatment being the best, but do not quantify the magnitude of differences between treatments. Several pairwise comparisons in this study showed a substantial overlap in 95% confidence intervals.

The present network meta-analysis had several limitations. First, the distribution of the included studies evaluating different VA regimens was uneven, with limited research available for the 7-day VA regimen and, within each regimen, the distribution of studies across different dosages was also imbalanced. Second, existing data reveal inconsistent findings regarding the eradication efficacy of the 10-day high-dose regimen across studies. some studies indicate that this regimen fails to achieve satisfactory eradication, whereas others deem it to be sufficient. This discrepancy may be related to regional variations in *H. pylori* resistance rates. Besides, we recommend that future trials adopt the CTCAE system for standardized adverse event grading and fully report the specific data for each severity level.

## 5 Conclusion

In summary, this network meta-analysis revealed that VA dual therapy was associated with higher eradication rates than BQT as a first-line treatment for *H. pylori* eradication. Considering both efficacy and safety, a 14-day low-dose VA regimen may offer a favorable balance between treatment duration and dosage among the regimens evaluated. This suggests that it may be a promising option for *H. pylori* eradication.

## Data Availability

The original contributions presented in the study are included in the article/Supplementary material, further inquiries can be directed to the corresponding author.
