# Supplementary material for: Comparative efficacy of different durations and dosages of vonoprazan and amoxicillin dual therapy: a network meta-analysis of randomized controlled trials
Source: Front Med (Lausanne). 2026 Aug 28;13:1853849. doi: 10.3389/fmed.2026.1853849 (PMC13563763; doi:10.3389/fmed.2026.1853849)
Supplement: Supplementary file 1 [file Supplementary_file_1.docx]

**Supplementary Figure Legend**

**Supplementary Figure 1 Risk of Bias Summary Plot on Percentage, Intention-to-Treat**

Green, yellow, and red segments represent low risk of bias, some concerns, and high risk of bias, respectively. The domains assessed include randomization process, deviations from intended interventions, missing outcome data, measurement of the outcome, selection of the reported result, and overall bias.

**Supplementary Figure 2 Cumulative rankogram on the probability of each treatment being at each rank**

Curves represent the probability distribution of each treatment achieving a specific rank, based on the network meta-analysis. VA14 had the highest probability of being the most effective treatment (rank 1), while BQT had the highest probability of being the least effective (rank 4).

**Supplementary Figure 3 Adherence comparisons across regimens**

Group B achieved a significantly higher adherence rate than BQT (*OR* = 1.54, *95% CrI* 1.02–2.39; *P*< 0.05). No statistically significant differences in adherence were observed among the different VA regimens (*P*＞ 0.05)

**Supplementary Figure 4 Eradication Rates of Group B vs. Group C**

Group B achieved a significantly higher *Helicobacter pylori* eradication rate than Group C (*OR*= 2.06, *95% CI* 1.29–3.28; *P*< 0.05). No heterogeneity was observed between studies (*P*> 0.05).

**Supplementary Figure 5 The comparison of different VA regimens and BQT in *Helicobacter pylori* eradication rates and adverse event rates, and different doses of VA regimens in *Helicobacter pylori* eradication rates and adverse event rates**

comparison-adjusted Funnel plots of standard error versus comparison-specific centered effect size are shown. The dashed lines represent the 95% confidence limits of the expected distribution. Symmetrical distribution of studies across all comparisons suggests no evidence of significant publication bias or small-study effects.

**Supplementary Figure 6 Adherence comparisons across regimens**

Trace (left) and posterior density (right) plots for MCMC convergence diagnostics of the network meta‑analysis. (A, B) Overall model; (C, D) high‑dose versus low‑dose subgroup model. All parameters showed good convergence, with well‑mixed chains and unimodal posterior densities.

**Supplementary Figure 7** **Gelman-Rubin shrink factor plots for convergence diagnosis**

(A, B) Overall network model; (C, D) high/low-dose subgroup model. All shrink factors converged close to 1, indicating satisfactory model convergence.

**Supplementary Figure 8** **leave-one-out sensitivity analysis**

Leave‑one‑out sensitivity analysis revealed that the initial inconsistency detected by node‑splitting was no longer present after excluding certain studies, with direct and indirect evidence becoming consistent.

**Supplementary Figure 9** **Sensitivity analysis using a fixed-effect model**

The difference in eradication rates between VA14 and BQT was statistically significant (*OR*=1.6, *95% CrI* 1.4–1.9), as was the difference between VA10 and BQT (*OR*=1.4, 95% *CrI* 1.1–1.7); the difference between VA7 and BQT remained non‑significant (*OR*=1.1, 95% CrI 0.74–1.5). All comparisons between the different VA regimens and BQT showed statistically significant differences in adverse event rates.
