# Supplementary material for: Comparative efficacy of different durations and dosages of vonoprazan and amoxicillin dual therapy: a network meta-analysis of randomized controlled trials
Source: Front Med (Lausanne). 2026 Aug 28;13:1853849. doi: 10.3389/fmed.2026.1853849 (PMC13563763; doi:10.3389/fmed.2026.1853849)
Supplement: Supplementary file 2 [file Table_1.docx]

**Supplementary Table 1 League table of *H. pylori* eradication rates.**

See footnote under Supplementary Table 3 for interpretation notes.

|  | A（VA14） | B（VA10） | C(VA7) | D(BQT) |
| --- | --- | --- | --- | --- |
| A（VA14） | A | 0.86 (0.64, 1.16) | 0.71 (0.43, 1.19) | 0.59 (0.47, 0.74) |
| B（VA10） | 1.16 (0.86, 1.57) | B | 0.83 (0.5, 1.37) | 0.69 (0.51, 0.93) |
| C（VA7） | 1.41 (0.84, 2.31) | 1.21 (0.73, 1.98) | C | 0.84 (0.5, 1.34) |
| D(BQT) | 1.69 (1.35, 2.12) | 1.45 (1.08, 1.98) | 1.2 (0.74, 1.98) | D |

**Supplementary Table 2 League table of adverse event incidence rates.**

Odds ratios (95% credible intervals) are presented. Treatments are ordered by SUCRA ranking. Values in the upper triangle represent the effect of the column treatment relative to the row treatment; values in the lower triangle represent the reciprocal. Statistically significant results (95% CrI does not include 1) are indicated in bold.

|  | A（VA14） | B（VA10） | C(VA7) | D(BQT) |
| --- | --- | --- | --- | --- |
| A（VA14） | A | 0.86 (0.64, 1.16) | 0.85 (0.52, 1.41) | 3.05 (2.49, 3.75) |
| B（VA10） | 1 (0.76, 1.31) | B | 0.85 (0.52, 1.38) | 3.03 (2.31, 4.02) |
| C（VA7） | 1.17 (0.71, 1.93) | 1.18 (0.73, 1.92) | C | 3.58 (2.21, 5.82) |
| D(BQT) | 0.33 (0.27, 0.4) | 0.33 (0.25, 0.43) | 0.28 (0.17, 0.45) | D |

**Supplementary Table 3 SUCRA values and P‑scores with ranking of different treatment regimens for *Helicobacter pylori* eradication rates and adverse event rates.**

For eradication rates, higher SUCRA and P‑score values indicate better efficacy. For adverse event rates, higher SUCRA and P‑score values indicate higher event rates.

|  | Eradication rate SUCRA (%) | Eradication rate P‑score | Adverse event incidence SUCRA (%) | Adverse event incidence P‑score |
| --- | --- | --- | --- | --- |
| A(VA14) | 91.7 | 0.92 | 59.34 | 0.59 |
| B(VA10) | 64.2 | 0.64 | 57.69 | 0.58 |
| C(VA7) | 35.9 | 0.36 | 82.98 | 0.83 |
| D(BQT) | 8.1 | 0.08 | 0.00 | 0.00 |
